# A Polyclonal Aptamer Library for the Specific Binding of the Gut Bacterium *Roseburia intestinalis* in Mixtures with Other Gut Microbiome Bacteria and Human Stool Samples

**DOI:** 10.3390/ijms23147744

**Published:** 2022-07-13

**Authors:** Hu Xing, Yiting Zhang, Markus Krämer, Ann-Kathrin Kissmann, Valerie Amann, Heinz Fabian Raber, Tanja Weil, Kai R. Stieger, Uwe Knippschild, Marius Henkel, Jakob Andersson, Frank Rosenau

**Affiliations:** 1 Institute of Pharmaceutical Biotechnology, Ulm University, Albert-Einstein-Allee 11, 89081 Ulm, Germany; hu.xing@uni-ulm.de (H.X.); yiting.zhang@uni-ulm.de (Y.Z.); markus-1.kraemer@uni-ulm.de (M.K.); valerie.amann@uni-ulm.de (V.A.); raber.heinz@gmx.de (H.F.R.); 2 Max-Planck-Institute for Polymer Research Mainz, Ackermannweg 10, 55128 Mainz, Germany; weil@mpip-mainz.mpg.de; 3 BIOMES NGS GmbH, Schwartzkopffstraße 1, Halle 21, 15745 Wildau, Germany; kai.stieger@biomes.world; 4 Department of General and Visceral Surgery, Surgery Center, Ulm University, Albert-Einstein-Allee 23, 89081 Ulm, Germany; uwe.knippschild@uniklinik-ulm.de; 5 Cellular Agriculture, TUM School of Life Sciences, Technical University of Munich, 85354 Freising, Germany; marius.henkel@tum.de; 6 AIT Austrian Institute of Technology, Giefinggasse 4, 1210 Vienna, Austria; jakob.andersson@ait.ac.at

**Keywords:** DNA aptamer, biosensor, in vitro diagnostic, *Roseburia intestinalis*

## Abstract

*Roseburia intestinalis* has received attention as a potential probiotic bacterium. Recent studies have demonstrated that changes in its intestinal abundance can cause various diseases, such as obesity, enteritis and atherosclerosis. Probiotic administration or fecal transplantation alter the structure of the intestinal flora, offering possibilities for the prevention and treatment of these diseases. However, current monitoring methods, such as 16S rRNA sequencing, are complex and costly and require specialized personnel to perform the tests, making it difficult to continuously monitor patients during treatment. Hence, the rapid and cost-effective quantification of intestinal bacteria has become an urgent problem to be solved. Aptamers are of emerging interest because their stability, low immunogenicity and ease of modification are attractive properties for a variety of applications. We report a FluCell-SELEX polyclonal aptamer library specific for *R. intestinalis* isolated after seven evolution rounds, that can bind and label this organism for fluorescence microscopy and binding assays. Moreover, *R. intestinalis* can be distinguished from other major intestinal bacteria in complex defined mixtures and in human stool samples. We believe that this preliminary evidence opens new avenues towards aptamer-based electronic biosensors as new powerful and inexpensive diagnostic tools for the relative quantitative monitoring of *R. intestinalis* in gut microbiomes.

## 1. Introduction

*R. intestinalis* was first isolated from human feces in 2002 [1]. It is an anaerobic, Gram-positive, slightly curved, butyrate-producing rod-shaped and motile bacterium [2], which provides multiple benefits to the host, including the production of the essential short-chain fatty acid (SCFA) butyrate [3]. Previous studies have demonstrated that butyrate plays a central role in cell differentiation and apoptosis as a signaling molecule, and induces the differentiation of Treg cells and interleukin (IL)-10-producing T cells, which significantly improves intestinal inflammation [4,5,6]. Inflammatory bowel disease (IBD) includes ulcerative colitis (UC) and Crohn’s disease (CD) [7,8]. In one study, the inflammation of ulcerative colitis was found to be significantly suppressed after flushing the colon with butyrate [9]. The use of mouse models further argues that butyrate inhibits the development of atherosclerosis [10,11]. As a significative butyrate producer, *R. intestinalis* is thus promising as a new probiotic for maintaining intestinal health and the treatment of diseases [5,12,13,14]. Earlier studies have also demonstrated a significant reduction in the abundance of *R. intestinalis* in the intestine of untreated UC and CD patients [15,16,17]. In a subsequent study, DSS-induced colitis in mice representing a human UC model and TNBS-induced colitis in mice representing a human CD model described that *R. intestinalis* significantly ameliorated DSS- and TNBS-induced colitis [18,19]. A significantly lower abundance of *R. intestinalis* was found in the intestine of patients with the constipated irritable bowel syndrome (C-IBS) than in the controls [20]. Later, Parthasarathy et al. sequenced 16S rRNA in the colonic mucosa and the fecal microbiota of constipated patients and healthy people, respectively, and found that *Roseburia* was closely associated with faster colonic transit [21]. An early study has demonstrated that butyrate can inhibit hepatic fat production and accumulation during hepatic metabolism [22]. By constructing a mouse model of alcohol-related liver diseases (ALDs), *Roseburia* supplementation was found to significantly improve the ALD symptoms by inhibiting hepatic inflammation and steatosis [23]. Subsequently, Pan et al. found that the number of butyrate-producing bacteria in the gut of patients with nonalcoholic fatty liver disease (NAFLD) was significantly reduced compared to the healthy population by analyzing the gut microbiome [24]. This further supports that *R. intestinalis* plays an essential role in maintaining intestinal health for the prevention and treatment of digestive diseases [2]. Recently, Yao et al. found that *Cyclocarya paliurus* (CCPP) could alleviate the symptoms of type 2 diabetes (T2DM) by increasing the production of short-chain fatty acids (SCFAs), represented by *R. intestinalis*, and promoting SCFA production through the construction of T2DM [25]. An assessment of the gut microbiota in patients with type 1 diabetes mellitus (T1DM) by Leiva-Gea et al. reported a lower abundance of *R. intestinalis* in the gut than in healthy individuals [26]. In another study, the analysis of gut microbiota from 40 monozygotic twin pairs revealed a positive correlation between *R. intestinalis* and body mass index (BMI) [27], suggesting a significant correlation between changes in *R. intestinalis* abundance in the gut and obesity. The study of cancer-associated microorganisms is also crucial in the cancer research process [2]. There is evidence that *R. intestinalis* abundance is much lower in the gut of colorectal cancer (CRC) patients than in normal subjects, making it a reliable novel biomarker in CRC [28,29].

**Figure 1:**
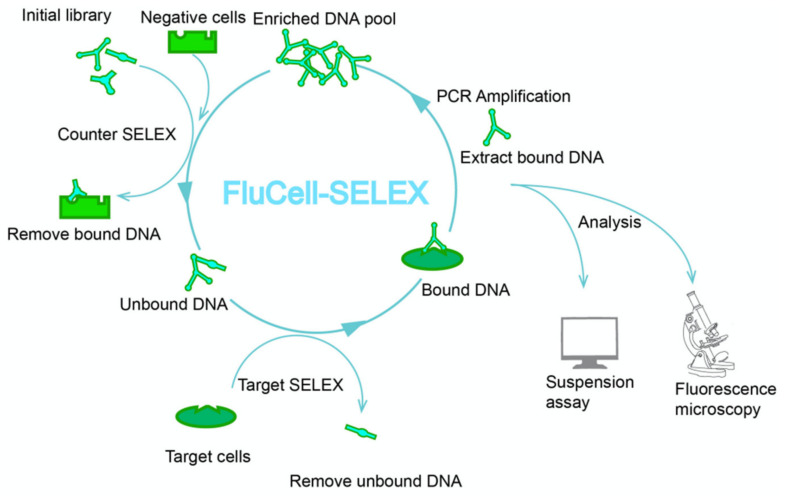
Directed evolution of a polyclonal aptamer library using FluCell-SELEX, which identifies *R. intestinalis* explicitly. Counter SELEX can focus the initial library to remove aptamers linked to control cells, a mixture of *Akkermannsia muciniphila*, *Allobaculum stercoricanis*, *Blautia producta*, *Parabcteroides distasonis* and *Rikenella microfusus*. This process was implemented to increase selection pressure to improve efficiency of the molecular evolution process. The unbound aptamers were then further co-incubated with *R. intestinalis* in the Target SELEX to obtain aptamers that could bind to the dedicated target cells. This process is subsequently repeated several times with increasing harshness of the binding conditions to obtain aptamer libraries that specifically bind to *R. intestinalis* cells with high affinity. The aptamer libraries obtained after each round of selection were analyzed by fluorometric assays in suspension and fluorescence microscopy to determine their affinity and specificity.

Electronic biosensors are easy to handle, often portable, and, in principle, can analyze in real-time, making them suited for measuring biomarkers [30]. In recent years, aptamer-based biosensors have received much attention for their excellent performance in terms of sensitivity, stability and selectivity [31,32]. Specific aptamers usually originate from libraries of single-stranded RNA or single-stranded DNA fragments obtained by the systematic evolution of ligands by exponential enrichment (SELEX) technology. The aptamers can be evolved towards a high specificity and affinity for target molecules, such as proteins, amino acids, viruses, pathogens and higher cells and tissues [31,33,34,35,36,37,38,39,40]. Initial random libraries consist of 10^12^–10^16^ single-stranded deoxyribonucleic acid (ssDNA) molecules and each sequence contains fixed primer binding sites and a random region in the center of the molecule. After incubation of the random library with the target cells during the screening process, the DNA sequences bound to the target cells are amplified by PCR, and the next round of screening is performed [31,36]. Based on our previous studies, we systematically evolved a library of Cyanine 5 (Cy5) fluorescently labeled DNA aptamers that bind specifically to the target cells using a technique called FluCell-SELEX. We were able to not only demonstrate the evolution of binding specificity by following the increase in fluorescence along the SELEX rounds, but also show that the resulting libraries (called “polyclonal” or “focused” libraries) could outperform the individual aptamers isolated from these libraries after traditional aptamer characterization via Next Generation Sequencing (NGS) and comprehensive bioinformatic analyses [35,36]. The use of such polyclonal libraries represents a considerable simplification of the generation of specific binding entities for biological assays and for the construction of electronic biosensors, and has proved its potential in sensors for the quantification of isoforms of a health-relevant blood protein [40]. To omit all of the sequencing and analysis efforts represents another advantage of already using polyclonal libraries without the isolation of individual aptamers, regarding the possible speed of the technological development of functional and specific binding entities; this then allows a fast reaction in case of emerging health-threats, such as novel pathogens, to equip health systems with adequate sensing techniques. Here we describe the use of the FluCell-SELEX (Figure 1) to evolve an aptamer library that can bind *R. intestinalis* cells with high affinity, allowing for the specific labeling of cells for fluorescence microscopy, binding assays and to distinguish *R. intestinalis* from other gut bacteria. The initial experiments demonstrate that measurements will be possible in complex mixtures and stool samples, which suggests the *R. intestinalis* polyclonal aptamer library presented here is a promising binding entity for the construction of electronic biosensors. To our knowledge, we are the first to develop this specific DNA aptamer library for the labeling of this highly promising auspicious probiotic bacterium, to pave the way for the future development of biosensors for the specific detection of *R. intestinalis*.

## 2. Results

### Aptamer Libraries Specific for R. intestinalis

**Figure 2:**
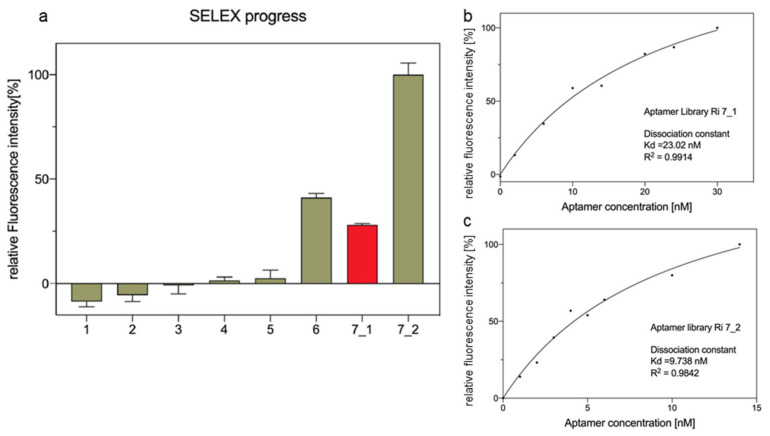
(**a**) Binding analysis of polyclonal aptamer library selectively targeting *R. intestinalis*. The progressively increasing fluorescence signal shows the enrichment of the aptamer library recognizing *R. intestinalis* (green-labeled). A different screening strategy was used in the seventh round of screening, and the aptamer library Ri 7_2 (green-labeled) showed a significantly enhanced ability to bind to *R. intestinalis* compared to the aptamer library Ri 7_1 (red-labeled). All experiments were conducted in triplicates (*n* = 3); (**b**) The dissociation constant (K_d_) of the polyclonal aptamer library Ri 7_1 was calculated as 23.02 nM, and the deviation of the coefficient of determination (R^2^) was 0.9914; (**c**) The dissociation constant (K_d_) of the polyclonal aptamer library Ri 7_2 was calculated as 9.738 nM, and the deviation of the coefficient of determination (R^2^) was 0.9842.

The FluCell-SELEX process was performed for seven rounds to obtain the functional library, which was able to recognize *R. intestinalis*. The labeling efficiency developed slowly with the respective libraries from round one to round five, being clearly below the detection limit, in the later stages of the SELEX it increased from round five to seven, while introducing counter-selection with mixtures of bacteria in round six. Interestingly, the labeling was impaired for the round seven library (designated as round 7_1 in Figure 2a) compared to round six. We thus tentatively attempted a modification of the counter-selection procedure, in a way that tenfold more unspecific target cells were offered in the alternative round 7_2. In fact, this led to a considerable increase in the *R. intestinalis* labeling and subsequently binding constants were determined. While a gradual increase in the fluorescence intensity was observed when fixed amounts of the *R. intestinalis* were co-incubated with different concentrations of the same aptamer library, the curve fitted with a typical one-site-specific binding model revealed that the aptamer library Ri 7_2 exhibited a reasonable low K_d_ value of 9.74 nM. In contrast, the dissociation constant of Ri 7_1 was only 23.02 nM, demonstrating a significantly improved sensitivity of the Ri 7_2 library, respectively (Figure 2b,c). Reducing (at a given and constant concentration of aptamers) the sequence space, and thus the amount of non-specific aptamers, increases at the same time the relative amount of specific aptamers in the library. This in turn leads to an increase in specificity and an increase in binding and the observed reduction in the dissociation constant K_d_.

**Figure 3:**
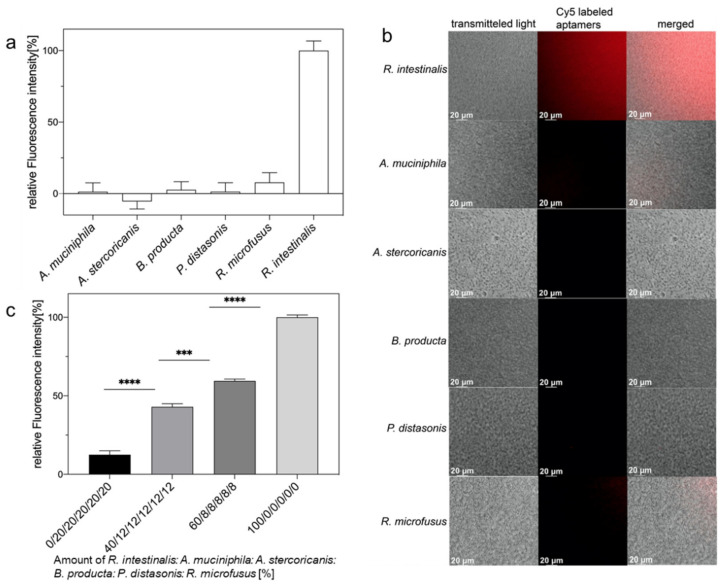
(**a**) Ri 7_2 specificity analysis of polyclonal aptamer library. The aptamer library was incubated with *A. muciniphila*, *A. stercoricanis*, *B. producta*, *P. distasonis*, *R. microfusus* and *R. intestinalis*, respectively. All experiments were performed with 10^8^ cells and 5 pmol aptamers in triplicates (*n* = 3). Aptamers bound to *R. intestinalis* were significantly higher than the other five intestinal bacteria; (**b**) Fluorescence microscopy of the polyclonal aptamer library Ri 7_2 labeled with *R. intestinalis*. The binding of the Cy5-labeled polyclonal aptamer library to *R. intestinalis* showed the strongest fluorescent signal. The other five gut bacteria served as controls and only showed weak fluorescence signals; (**c**) Detection of increasing amounts of *R. intestinalis* by the fluorescent-labeled polyclonal aptamer library Ri 7_2 in a mixture of intestinal bacteria including *A. muciniphila*, *A. stercoricanis*, *B. producta*, *P. distasonis* and *R. microfusus*, which were adjusted in equal optical densities and mixed at different ratios. Scale bars represent 20 µm. All experiments were performed with 10^8^ cells and 5 pmol aptamers in triplicates (*n* = 3). *p* values < 0.05 were considered significant. *** denotes *p* < 0.001 and **** *p* < 0.0001.

Based on the experience of previous studies, we tentatively suspected that after round seven of evolution, the aptamer library Ri 7_2 had met the criteria of a sufficient affinity and specificity for the detection of *R. intestinalis* in different assays. Here, we used 10^8^ CFU each of *A. muciniphila*, *A. stercoricanis*, *B. producta*, *P. distasonis*, *R. microfusus* and *R. intestinalis* and 5 pmol of the aptamer library Ri 7_2, to determine the labeling specificity (Figure 3a). The results showed that the binding of the Cy5-labeled polyclonal aptamer library to the dedicated target *R. intestinalis* showed the strongest fluorescence signal, whereas binding to the other five gut bacteria serving as controls was only marginal (Figure 3a). The ability of the aptamer library Ri 7_2 to discriminate *R. intestinalis* from a single control bacterium was verified by a labeling experiment, which used fluorescence microscopy of the individual bacterial strains. The results showed that the aptamer library Ri 7_2 specifically showed intense labeling of *R. intestinalis*, but ultimately failed to label the other five control bacteria (Figure 3b). Next, we adjusted *R. intestinalis* and five other intestinal bacteria, *A. muciniphila*, *A. stercoricanis*, *B. producta*, *P. distasonis* and *R. microfusus* to the same optical density and mixed them at different ratios. The final fluorescence labeling experiments showed that the increasing number of *R. intestinalis* in this series of samples could be tracked perfectly, and the fluorescence intensity showed a clear linear relationship with the number of *R. intestinalis* (Figure 3c). This shows that already with the Ri 7_2 polyclonal library labeling applications can be performed with adequate specificity and accuracy.

**Figure 4:**
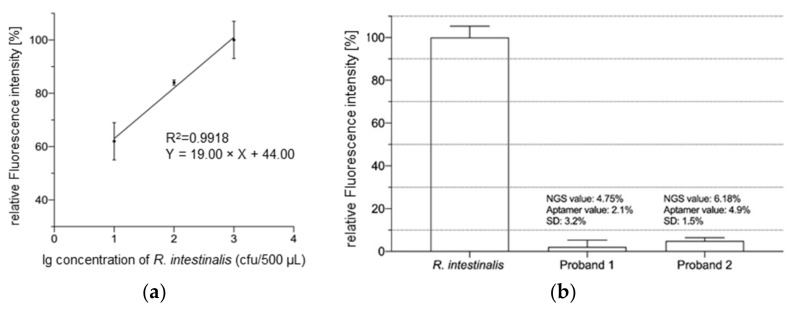
(**a**) Sensitivity determination of aptamer library Ri 7_2. In a 500 µL reaction system containing 5 pmol aptamer library Ri 7_2, the relative fluorescence intensity showed a positive correlation with the cell number when the number of *R. intestinalis* varied within 10–10^3^ CFU. A clear positive correlation was observed (R^2^ = 0.9918), while the detection limit of the aptamer library was 10 CFU at this time; (**b**) *Roseburia* abundance in fecal samples based on the aptamer library Ri 7_2 and 16s rRNA NGS (see Tables S2 and S3, Supplementary Materials). The “NGS value” represents the actual *Roseburia* content in fecal bacteria as determined by NGS. The “Aptamer-value” shows the *Roseburia* abundance in fecal bacteria as determined by the aptamer library Ri 7_2. The SD represents the standard deviation during the measurement of the target value.

To further quantify the sensitivity of the aptamer library Ri 7_2 in the fluorometric assay, we successively reduced the number of *R. intestinalis* cells in the system and thus generated a calibration curve (Figure 4a). The concentration of *R. intestinalis* showed a significant linear relationship with the fluorescence intensity (R^2^ = 0.9918), when the number of bacteria in the 500 µL reaction system was varied from 10^1^ to 10^3^ CFU. The experimental detection limit of *R. intestinalis* for the aptamer library Ri 7_2 was 10^1^ CFU. Thus, the high sensitivity of the aptamer library Ri 7_2 for identifying *R. intestinalis* also provides the possibility of detecting trace amounts of *R. intestinalis* in the fecal bacteria. Finally, to further validate the potential of the aptamer library Ri 7_2 for practical applications, we first obtained the exact amount of *Roseburia* in the feces of two healthy volunteers (proband 1 and proband 2 in Figure 4b) by 16S rRNA next-generation sequencing. We then used 500 µL samples containing 5 pmol of the aptamer library Ri 7_2 mixed with *R. intestinalis* (10^8^ CFU) or fecal bacteria (10^8^ CFU) for the binding assay. The relative fluorescence intensity of *R. intestinalis* as a control group was 100%. In contrast, the relative fluorescence intensity of the experimental group represented the relative abundance of *Roseburia* in the fecal bacteria. We obtained 4.75% and 6.18% for proband 1 and proband 2, respectively, of *Roseburia* in the fecal bacteria of the probands by NGS (see Tables S2 and S3, Supplementary Materials). The results originating from the fluorometric assay performed in parallel with the same samples were in the same range and, with 2.1% and 4.9%, reflected the higher cell number of *R. intestinalis* in the stool sample of proband 2 (Figure 4b). The standard deviations of these two datasets were 3.2% and 1.5%, respectively. Considering the possible experimental errors, it is reasonable to believe that the aptamer library Ri 7_2 is feasible and reliable for detecting the abundance of *Roseburia* in feces in practice.

## 3. Discussion

Since the first application of the SELEX technology in 1990, a large number of aptamer libraries for different target molecules have been developed [41,42], including simple targets, such as inorganic and organic small molecules, proteins, etc., and complex targets, such as whole cells or organisms [43]. Directing aptamer evolution against native proteins presented in the living cell may represent not only a simplification but also an improvement of the method, because this omits aptamer screening against the recombinant proteins with potentially non-natural conformations or insufficient purity, especially of the transmembrane proteins [44,45,46]. Unlike other SELEX methods, Cell-SELEX is a whole-cell aptamer evolution process. Thus, the target molecules on the cell surface have their natural conformation, and the entire process is performed without prior knowledge of the molecular targets on the cell surface [47,48]. Thus, Cell-SELEX also minimizes the risk that the evolved aptamer library will only bind to purified proteins and not recognize proteins with natural conformations on the living cells. At the same time, the single aptamer in the evolved polyclonal aptamer library by Cell-SELEX can bind with different affinities to different target sites on the surface of the target cell membrane, in which case the final aptamer library has a different level of complexity, due to the significant differences in the number and abundance of potential target molecules [44,46]. The good labeling flexibility and assay fidelity of the polyclonal aptamer libraries compared to single aptamers, as well as the simultaneous binding of multiple potential targets further reduce the signal fluctuations caused by single aptamers, resulting in more reliable assays [36]. Cell-SELEX, in combination with the polyclonal library strategy, thus may represent a promising technology in many fields, ranging from medical diagnostics and sensor development to new therapeutic concepts including cancer therapy [49].

Microbiome NGS analysis involves the sequencing of 16S rRNA genes, that are highly conserved in prokaryotes and can thus be amplified even from yet unknown organisms [50]. However, obtaining reliable species-level or even genus-level classification is difficult, due to the lack of partitioning ability of the sequenced marker genes [51], since the 16S rRNA genes, although they contain nine taxonomically distinguishable hypervariable regions, do not have a single hypervariable region that can distinguish between all of the species [52]. This feature makes it difficult to distinguish different bacteria of the same genus by NGS. This represents the major principal drawback of 16S rRNA sequencing, resulting in an overestimation of individual species, which are in fact principally not counted as a species. The polyclonal libraries presented in this study originating from the FluCell-SELEX against *R. intestinalis* were characterized and found to increase their specificity up to the final round, 7_2. As described for the libraries from previous projects [31,35,36,40], this library Ri 7_2 was suitable for the labeling of *R. intestinalis* in fluorescence-based microtiter plate assays and for fluorescence microscopy. The measurements were also performed as microbiome samples derived from the stool samples of two human probands and compared to measurements of sequence abundance via NGS. In contrast to the 16S rRNA derived genome counts of 4.75% and 6.18%, for *Roseburia* the relative numbers determined by the calibrated fluorometric aptamer analysis were in consequence lower (2.1% and 4.9%). Interestingly, the tendency that proband 2 had more *Roseburia* species was also visible in the aptamer analysis, while the relative difference between both of the analysis techniques was 55.8% lower in the aptamer experiment for proband 1 compared to the NGS value, and 20.7% for proband 2, respectively. Without yet having determined the binding affinity of library Ri 7_2 against other non-*intestinalis Roseburia* species, these results suggest that Ri 7_2 meets the expectation that binding occurs mainly below the genus level. This may not only open the opportunity to develop novel sub-genus detection assays for *R. intestinalis,* but also in turn, with additional dedicated libraries, for other *Roseburia* species. We have recently developed a graphene field-effect transistor-based sensor chip for the quantification and differentiation of a biological marker involving polyclonal aptamer libraries [40]. This technology, in combination with the *R. intestinalis* polyclonal aptamer library as a binding entity on such a chip, may allow in the near future the development of sensitive and cost-efficient monitoring devices for the rapid and easy supervision of e.g., probiotic supplementation studies with *R. intestinalis,* and also in a generalized form for all other health-relevant gut bacteria.

## 4. Materials and Methods

### 4.1. Cell Lines and Cell Culture

The bacteria strains *R. intestinalis* (DSM-14610), *P. distasonis* (DSM-29491), *A. muciniphila* mucT (DSM-22959), *A. stercoricanis* (DSM-13633), *B. producta* (DSM-29491) and *R. microfusus* (DSM-15922) were cultivated in Schaedler Broth Medium at 37 °C under anaerobic conditions.

### 4.2. Single-Stranded DNA (ssDNA) Library and Primers

The random sequence library was synthesized and purified (TriLink BioTechnologies, Inc, San Diego, CA, USA). The sequence was as follows: 5′-TAGGGAAGAGAAGGACATATGAT-N_(40)_-TTGACTAGTACATGACCACTTGA-3′. The initial library consisted of three parts. The random part contained a randomly varying central region composed of 40 nucleotides and two fixed sequences of 23 nucleotides each, which could be precisely and complementarily bound to the primers. The other parts were comprised of Cyanine 5-labeled forward primer (Cy5-P_1_) 5′-Cy5-TAGGGAAGAGAAGGACATATGAT-3′, and phosphate-labeled reverse primer (Phosphate-P_2_) 5′-Phosphate-TCAAGTGGTCATGTACTAGTCAA-3′ (Eurofins Genomics, Ebersberg, Germany).

### 4.3. Cell-SELEX

The Cell-SELEX included counter SELEX as well as target SELEX. In this chapter, the control bacteria used in counter SELEX were *A. muciniphila* mucT, *A. steroricanis*, *P. distasonis*, *B. producta* and *R. microfusus*. The aptamers that did not bind to the control bacteria were obtained, which could be further screened by target SELEX to gain an aptamer library specifically targeting *R. intestinalis*. The Cell-SELEX could be divided into the following steps.

#### 4.3.1. Cell Pretreatment

The cells were incubated under anaerobic conditions for 21–24 h and subsequently centrifuged at 6000 rpm for 1 min. They were then washed three times with 1× DPBS buffer. Finally, the OD_600_ of the bacterial solution was adjusted to 1.

#### 4.3.2. Aptamer Library Activation

A total of 0.5 nmol of the original library or 10/5 pmol ssDNA library were added to 500 µL 1× DPBS and incubated at 95 °C for 5 min, ice bath for 5 min, and then left at room temperature for 20 min to ensure that the aptamers had the same 3D structure.

#### 4.3.3. Screening

The activated library was incubated with control cells for 1 h at 37 °C and centrifuged at 3000 rpm for 2 min and the pellet was discarded. To the remaining aptamer library, BSA (100 mg/mL) and tRNA (10 mg/mL) with increasing amounts were added to increase the stringency and then incubated with *R. intestinalis* at 37 °C. The supernatant was then removed by centrifugation at 3000 rpm for 2 min, and finally, the pellet was washed with 1× DPBS to discard unbound nucleic acids (see Table S1, Supplementary Materials).

#### 4.3.4. Elution

The cells from the previous step were resuspended in 100 µL 1× DPBS and incubated at 95 °C for 5 min to disrupt the aptamer 3D structure and separate the cells from the aptamer. The aptamer bound to *R. intestinalis* was collected by subsequent centrifugation at 11,000 rpm for 1 min.

#### 4.3.5. Acquisition of Secondary Libraries

The collected aptamers were amplified by PCR. The amplification conditions were as follows: 2 min at 80 °C; 2 min at 85 °C; 2 min at 90 °C; 3 min at 94 °C; followed by 25 cycles of 30 s at 94 °C; 30 s at 56 °C; 10 s at 72 °C; then 2 min at 72 °C. Subsequently, the PCR product was purified (MACHEREY-NAGEL GmbH & Co. KG, Düren, Germany) and then decomposed into single-stranded DNA by lambda-exonuclease catalysis (New England Biolabs, Ipswich, MA, USA). The treated sample was finally purified by an optimized PCR clean-up kit (MACHEREY-NAGEL GmbH & Co. KG, Düren, Germany) to obtain the new DNA pool for the following selection rounds, in which the required binding buffer was added with 1.5 volumes of isopropanol and 10 µL of NaAc solution (pH 5).

#### 4.3.6. Binding Assay

The *R. intestinalis* was preprocessed by the above method (see Section 4.3.1). The binding affinity of the aptamer library was determined by incubating *R. intestinalis* (1 mL OD_600_ = 1) with 5 pmol of the activated Cy5-labeled aptamer library in 500 µL of 1× DPBS for 30 min at 37 °C. Subsequently, the supernatant was removed by centrifugation at 3000 rpm for 2 min, and the pellet was resuspended in 100 µL of 1× DPBS buffer after washing three times to obtain the cell-bound aptamers after elution. Finally, the fluorescence intensity to determine the screening status was measured using an Infinite M200 spectrophotometer (TECAN, Männedorf, Switzerland) at an excitation wavelength of 637 nm and an emission of 670 nm.

### 4.4. Determination of High Specificity Aptamer Libraries

#### 4.4.1. Semi-Quantitative Analysis of *R. intestinalis*

All of the bacterial solutions were preprocessed by the above method (see Section 4.3.1), and then the *R. intestinalis* and the control strains, including other five intestinal bacterium *A. muciniphila* mucT, *A. steroricanis*, *P. distasonis*, *B. producta* and *R. microfusus*, were mixed in different ratios. The ratios of the control strains and *R. intestinalis* were reduced from 0:1 (*R. intestinalis*: control strains) to 1:0 (*R. intestinalis*: control strains). The semi-quantitative analysis was determined by incubating the increasing amount of *R. intestinalis* with 5 pmol of activated Cy5-labeled aptamer library in 500 µL of 1× DPBS for 30 min at 37 °C. Each group of samples was postprocessed, as described above (see Section 4.3). Finally, the amount of *R. intestinalis* was analyzed by comparing the fluorescence intensity of each experimental group and PBS as the control group.

#### 4.4.2. Affinity Analysis

The bacterial solution of *R. intestinalis* was preprocessed by the method described above (see Section 4.3.1). The binding affinity of the selected aptamer library was determined by incubating *R. intestinalis* (1mL OD_600_ = 1) with varying concentrations of aptamer candidates in 500 µL of 1× DPBS for 30 min at 37 °C. Finally, the dissociation constants (K_d_) of the fluorescent aptamers were determined by fitting the dependence of the fluorescence intensity on the concentration of the aptamers to the equation Y = B_max_ × X/(K_d_ + X) using GraphPad PRISM 8 (GraphPad Software, San Diego, CA, USA), with Y = the measured fluorescence, B_max_ = the maximal fluorescence and X = concentration of the aptamers.

#### 4.4.3. Fluorescence Microscopy

The bacterial solution of *R. intestinalis*, *A. muciniphila* mucT, *A. steroricanis*, *P. distasonis*, *B. producta* and *R. microfusus* were pre-treated by the above method (see Section 4.3.1). A total of 5 pmol of the aptamer library in 500 µL of 1× DPBS were activated as described earlier (see Section 4.3.2). Subsequently, the libraries were incubated with each bacterium (1 mL OD_600_ = 1) for 30 min at 37 °C. After centrifugation at 3000 rpm for 2 min, the supernatant was removed. The pellet was washed once with 500 µL of 1× DPBS and then resuspended in 500 µL of 1× DPBS. Afterwards, 100 µL of each bacterial mixture was transferred to a 96-well microplate. Finally, the fluorescence imaging of each group was obtained using fluorescence microscopy, which was performed using a Leica DMi8 coded (Leica Microsystems CMS GmbH, Wetzlar, Germany) at ×40 magnitude under transmitted light with the Y5 filter (excitation: 590–650 nm and emission: 662–738 nm).

### 4.5. Analysis of *R. intestinalis* Abundance in Human Samples

#### 4.5.1. Human Samples

The fecal samples from two lean healthy volunteers were used in this study. Volunteers were recruited from Ulm University and signed a written informed consent form. Permission was received from the local Ethics Committee of Ulm University (no. 30/20). In addition, the study was designed and conducted following the regulations governing the use of human study participants and in strict accordance with the standards set by the Declaration of Helsinki.

#### 4.5.2. Stool Bacteria Extraction

The stool samples were weighed, added to extraction buffer (1× DPBS), and vortexed for 1 min until no fecal pellets were visible. The fecal pellets were then removed by filtration. The filtrate was centrifuged at 6000 rpm for 1 min, followed by three washes with 1× DPBS buffer, and finally adjusted to the OD_600_ of the bacterial solution of one.

#### 4.5.3. Analysis Based on NGS

The fecal samples were collected using INTEST.pro (Biomes Laboratory, Wildau, Germany) and then measured and analyzed by BIOMES laboratory (Wildau, Germany), using 16s rRNA NGS for fecal bacterial abundance, according to Lilja et al., 2021 [53].

#### 4.5.4. Analysis Based on the Aptamer Library R.i 7_2

As described above, the *R. intestinalis* (see Section 4.3.1) and stool samples (see Section 4.5.2) were pretreated. Subsequently, 500 µL 1× DPBS containing 5 pmol of activated aptamer library was incubated with *R. intestinalis* (10^8^ CFU) and fecal bacteria (10^8^ CFU) (see Section 4.3.2), respectively, for 30 min at 37 °C, and the fluorescence intensity was measured after elution.

## 5. Conclusions

In summary, we provide the first aptamer library as a high-affinity binding entity with specificity towards *R. intestinalis*. We have described the possibility of using these aptamers in various diagnostic methods and demonstrated that the specific library Ri 7_2 retains its binding function towards this important member of the gut microbiome, even in binding analyses using human stool samples. Without also having yet identified single aptamers as the target structures on the cell membrane, we believe that this opens new avenues for the rapid and reliable diagnosis of *R. intestinalis* in a new generation of biosensors in the future.

## Data Availability

The data can be found online or from authors for valid reasons.

## Supplementary Materials

The following supporting information can be downloaded at: https://www.mdpi.com/article/10.3390/ijms23147744/s1.
