# Supplementary material for: A Polyclonal Aptamer Library for the Specific Binding of the Gut Bacterium Roseburia intestinalis in Mixtures with Other Gut Microbiome Bacteria and Human Stool Samples
Source: Int J Mol Sci. 2022 Jul 13;23(14):7744. doi: 10.3390/ijms23147744 (PMC9317077; doi:10.3390/ijms23147744)
Supplement: Supplementary file 1 [file ijms-23-07744-s001.zip › ijms-1807830-supplementary.pdf]

Supplementary Materials:

Table S1. The selection conditions. The amount of aptamer library, counter SELEX, target SELEX, incubation temperature and incubation time, washing condition, and the amount of BSA/tRNA are indicated here.

SELEX rounds	Aptamer [pmol]	Counter SELEX (Incubation temperature and time）	Target SELEX (Incubation temperature and time）	Wash steps	BSA/tRNA [pmol]
1	500	-	250 µL OD_{600}=1<br>Incubation：37 ℃ for 1 h	1	600
2	10	-	250 µL OD_{600}=1<br>Incubation：37 ℃ for 1 h	1	900
3	10	-	250 µL OD_{600}=1<br>Incubation：37 ℃ for 1 h	2	1200
4	10	-	250 µL OD_{600}=1<br>Incubation：37 ℃ for 1 h	2	1500
5	10	-	250 µL OD_{600}=1<br>Incubation：37 ℃ for 1 h	3	1800
6	10	50 µL OD_{600}=2 per bacterium<br>Incubation：37 ℃ for 1 h	250 µL OD_{600}=1<br>Incubation：37 ℃ for 1 h	4	2100
7_1	5	50 µL OD_{600}=2 per bacterium<br>Incubation：37 ℃ for 1 h	250 µL OD_{600}=1<br>Incubation：37 ℃ for 1 h	6	2400
7_2	5	500 µL OD_{600}=2 per bacterium<br>Incubation：37 ℃ for 1 h	250 µL OD_{600}=1<br>Incubation：37 ℃ for 1 h	6	2400

Note: 1. To avoid non-specific interaction between the aptamer and the cell surface, the BSA (100 mg/mL) and tRNA (10 mg/mL) as competitors were incubated with targeted cells. 2. Counter-selection: Aptamer library was incubated with *A. muciniphila* mucT, *A. steroricanis*, *B. producta*, *P. distasonis*, and *R. microfusus*. 3. Target SELEX: Aptamer library was incubated with *R. intestinalis*.

Table S2. *Roseburia* abundance in fecal samples of proband 1 determined by 16S rRNA NGS.

**kingdom**	**phylum**	**class**	**order**	**family**	**genus**	**species**	**absolute_counts**	**relative_counts**
Bacteria	Firmicutes	Clostridia	Lachnospirales	Lachnospiraceae	Roseburia	hominis A2-183	2513	5,47303E+15
Bacteria	Firmicutes	Clostridia	Lachnospirales	Lachnospiraceae	Roseburia	unspecific_Roseburia	1,93166E+16	4,20693E+16

Table S3. *Roseburia* abundance in fecal samples of proband 2 determined by 16S rRNA NGS.

kingdom	phylum	class	order	family	genus	species	absolute_counts	relative_counts
Bacteria	Firmicutes	Clostridia	Lachnospirales	Lachnospiraceae	Roseburia	bacterium DJF.RR13	10	1,17518E+16
Bacteria	Firmicutes	Clostridia	Lachnospirales	Lachnospiraceae	Roseburia	hominis A2-183	22853	2,68563E+15
Bacteria	Firmicutes	Clostridia	Lachnospirales	Lachnospiraceae	Roseburia	unspecific_Roseburia	5,03133E+15	5,91271E+15
